# Circulating miR-147b as a diagnostic marker for patients with bacterial sepsis and septic shock

**DOI:** 10.1371/journal.pone.0261228

**Published:** 2021-12-16

**Authors:** Ngo Tat Trung, Tran Thi Lien, Vu Viet Sang, Nghiem Xuan Hoan, Nguyen Dang Manh, Nguyen Sy Thau, Dao Thanh Quyen, Tran Thi Thu Hien, Phan Quoc Hoan, Mai Hong Bang, Thirumalaisamy P. Velavan, Le Huu Song

**Affiliations:** 1 Centre for Genetics Consultation and Cancer Screening, Hanoi, Vietnam; 2 Vietnamese-German Center for Medical Research, Hanoi, Vietnam; 3 Department of Molecular Biology, Hanoi, Vietnam; 4 Institute of Clinical Infectious Diseases, Hanoi, Vietnam; 5 Department of Gastroenterology, Hanoi, Vietnam; 6 Institute of Tropical Medicine, Universitätsklinikum Tübingen, Tübingen, Germany; Oregon State University, UNITED STATES

## Abstract

**Background:**

Early diagnosis, precise antimicrobial treatment and subsequent patient stratification can improve sepsis outcomes. Circulating biomarkers such as plasma microRNAs (miRNAs) have proven to be surrogates for diagnosis, severity and case management of infections. The expression of four selected miRNAs (miR-146-3p, miR-147b, miR-155 and miR-223) was validated for their prognostic and diagnostic potential in a clinically defined cohort of patients with sepsis and septic shock.

**Methods:**

The expression of plasma miRNAs was quantified by quantitative PCR (qPCR) in patients with bacterial sepsis (n = 78), in patients with septic shock (n = 52) and in patients with dengue haemorrhagic fever (DHF; n = 69) and in healthy controls (n = 82).

**Results:**

The expression of studied miRNA was significantly increased in patients with bacterial sepsis and septic shock. The plasma miR-147b was able to differentiate bacterial sepsis from non-sepsis and septic shock (AUC = 0.77 and 0.8, respectively, p≤ 0.05), while the combination of plasma miR-147b and procalcitonin (PCT) predicted septic shock (AUC = 0.86, p≤ 0.05).

**Conclusions:**

The plasma miR-147b may be an useful biomarker independently or in combination with PCT to support clinical diagnosis of sepsis and equally prognosis of patients with septic shock.

## Introduction

Bacterial sepsis is a life-threatening condition in which bacteria enter the bloodstream and evoke a systemic inflammatory response unfolding a cascade of physiological changes leading to multiple organ failure [1]. There are three stages: sepsis, severe sepsis, and septic shock, the latter leading to organ dysfunction involving the cardiovascular system. The majority of sepsis and sepsis resulted deaths are reported from low and middle-income countries (LMIC) [2]. With a significant increase in antimicrobial resistance (AMR) especially in LMICs, sepsis management and treatment are increasingly challenging.

Early diagnosis, accurate antimicrobial treatment and subsequent patient stratification can improve sepsis outcome. Although inflammatory markers such as C-reactive protein (CRP) and procalcitonin (PCT) are useful biomarkers, blood cultures are commonly used in clinical practice as a diagnostic tool to identify etiologies. Recent advances in the use of automated systems with increased sensitivity and improved culture media facilitate rapid detection of microbial growth, but positive rates in sepsis patients vary depending on disease severity. While blood culture has its own intrinsic limitations, such as being laborious and only identifying microbes that grow under optimized culture conditions, polymerase chain reaction (PCR)-based method has the potential to fill this diagnostic gap [3].

Despite inflammatory markers and available diagnostic tools, a significant proportion of patients still suffer from sepsis burden, as the etiology of sepsis remains unclear in clinical practice [4–6]. A recent meta-analysis shows that inflammatory cytokines are unable to distinguish patients with systemic inflammatory response syndrome (SIRS) caused by other, noninfectious diseases [7, 8]. In this context, circulating biomarkers such as MicroRNAs (miRNAs) are proven useful as surrogates for infection diagnosis, severity, and case management.

The miRNAs, small non-coding RNAs, are secreted from cells and released into the bloodstream during infection, inflammation, and sepsis [9]. Their presence in plasma and/or serum indicates the role of circulating (cell-free) miRNAs in pathogenesis and are believed to be key gene modulators of distinct inflammatory responses [10]. A number of miRNAs are differentially expressed in the peripheral blood of patients with bacterial sepsis [10–16]. Among those well characterized, miR-146-3p, miR-147b and miR-155 were involved in the activation of NF-κB signaling pathway, a key mediator of inflammatory responses [11, 12] and the miR-223 involved in the regulation of monocyte-macrophage differentiation, neutrophil recruitment, and pro-inflammatory responses, and is a key regulator of innate immunity [17].

In addition to existing diagnostic tools, we hypothesize that the aberrant expression of such inflammation stimulated miRNAs (miR-146-3p, miR-155, miR-147b, miR-223,) is associated with bacterial sepsis and can be used as a surrogate maker that has additional value in the diagnosis of bloodstream infections. In this study, we validated the expression of four selected miRNAs (miR-146-3p, miR-147b, miR-155 and miR-223) in a clinically defined cohort of patients with sepsis and compared the respective miRNA expression with healthy controls. Furthermore, the prognostic and diagnostic potential of these selected miRNAs are validated in a subset of patients with sepsis and septic shock using the respective miRNA expression levels and with PCT values.

## Materials and methods

### Ethics approval and consent to participate

The study was approved by the institutional review board and an Independent Ethics Committee of the 108 Military Central Hospital, Hanoi, Vietnam. Informed written consent was obtained from all study patients.

#### Patients and sampling

A total of 130 blood plasma samples from sepsis (n = 78), septic shock (n = 52) and dengue hemorrhagic fever (DHF; n = 69) were collected from patients admitted to the 108 Military Central Hospital in Hanoi, Vietnam, between 2017 and 2019. In addition, plasma of healthy controls (n = 82, healthy blood donors) were also included in this study. Patients were evaluated based on clinical symptoms, Sequential Organ Failure Assessment (SOFA) scores, biochemical parameters, white blood cell count (WBC), platelet count (PLT), procalcitonin (PCT), liver enzymes including total bilirubin, alanine aminotransferase (ALT), aspartate aminotransferase (AST), serum creatinine, and lactate levels into subgroups that had either sepsis or septic shock, based on guidelines from the third consensus definition of sepsis and septic shock (Sepsis-3) [1].

#### Biochemical and serological tests

The total and direct bilirubin, alanine aminotransferase (ALT), aspartate aminotransferase (AST), procalcitonin (PCT), serum creatinine and lactate levels were measured on an auto-analyzer (Beckman Coulter AU5800 –Singapore).

#### miRNA extraction and cDNA synthesis

Total RNA, including miRNAs, was isolated from 500 μl plasma with TRIzol and was reconstituted in 50 μl water treated with Diethyl Pyrocarbonate (DEPC). Approximately 100 ng of total RNA was used for reverse transcription (RT) by RevertAid First Strand cDNA Synthesis Kit (ThermoFisher Scientific Inc., Singapore) following the manufacturer’s instruction. Primers used for cDNA synthesis were designed according to stem-loop theory as described previously [18] and are provided in the Table 1.

**Table 1:** Primer sequences used for real-time PCR to quantify the studied microRNAs.

Studied miRNA	miR base Accession code	Stem-loop primer	Forward primer
miR-146-3p	MIMAT0004608	GTTGGCTCTGGTGCAGGGTCCGAGGTATTCGCACCAGAGCCAACCTGAAG	GGTGTGCCTCTGAAATTCAGTT
miR-147b-3p	MIMAT0004928	GTTGGCTCTGGTGCAGGGTCCGAGGTATTCGCACCAGAGCCAACTAGCAG	GTGTTGTGTGCGGAAATGC
miR-223-5p	MIMAT0004570	GTTGGCTCTGGTGCAGGGTCCGAGGTATTCGCACCAGAGCCAACAACTCA	TGGTTTTGGGGCGTGTATTTGACAA
miR-155	MIMAT0000646	GTTGGCTCTGGTGCAGGGTCCGAGGTATTCGCACCAGAGCCAACACCCCT	GTTGGGGTTAATGCTAATCGTGAT
	Universal reverse primer	GTGCAGGGTCCGAGGT	

#### Quantification of miRNA by real-time PCR

After reverse transcription, the real-time quantitative PCR (qPCR) was performed. In brief, reaction mixtures consisted of 10μl of 2x SYBR-Green I master mix (Applied Biosystems, Foster City, CA, USA), 5μl of cDNA preparation, 5pmol of miRNA universal reverse primer GTGCAGGGTCCGAGGT and 5pmol of forward primer specific for miRNA (Table 1). The qPCR reaction was performed using the Stratagene M3000p device (Stratagene, San Diego, CA, USA) with a pre-incubation step at 50°C for 15 minutes, initial denaturation at 95°C for 5 minutes, followed by 45 cycles of 95°C for 15 sec and 60°C for 60 seconds. The cycle threshold (Ct) values were recorded and analyzed according to the comparative Ct method [19], in which the Ct value of miRNA-16 was used as normalization factor as recommended [20].

### Statistical analysis

Statistical analyses were performed by R v3.5.2 (https://www.r-project.org/). Values were presented as either mean with standard deviation (SD), median with range where appropriate. The non-parametric Mann-Whitney U-test or Kruskal-Wallis tests were used to compare quantitative variables between different groups where appropriate. Receiver operating characteristic (ROC) curves were generated based on random forest models to evaluate the predictive value of single or combined miRNA panels in discriminating sepsis or septic shock from other clinical status (non-sepsis and sepsis without shock, respectively) by computation of the area under the ROC curve (AUC). The level of significance was set at a two-sided p-value of < 0.05.

## Results

### Clinical characteristics of the patients

In total 78 patients with sepsis and 52 patients with septic shock were identified by blood cultures and/or by Sepsis 3.0 standards as previously described (Table 2) [1]. The demographic characteristics such as age and gender, and clinical characteristics such as SOFA score, biochemical parameters, white blood cell count (WBC), platelet count (PLT), procalcitonin (PCT), liver enzymes including total bilirubin, alanine aminotransferase (ALT), aspartate aminotransferase (AST), serum creatinine, lactate levels in patients with sepsis and septic shock are summarised in Table 2. WBCs, and creatinine were significantly increased in patients with sepsis compared with SIRS, DHF or healthy controls (P<0.05). PCT, ALT, AST, creatinine and lactate levels were elevated in patients with septic shock, patients with elevated SOFA score and in patients who died (*P*<0.05, Table 2).

**Table 2:** Characteristics of study participants according to clinical presentation.

Baseline characteristics	HC (n = 82)	DHF (n = 69)	Sepsis (n = 78)	Septic shock (n = 52)	*P*
Age (years)	35.5 (20–82)	43.3 (20–89)	55.65 ± (18.4)	56.89 ± (16.2)	* 0.97 **0.000
Gender (Male)	58 (69.9%)	26 (45.6%)	69 (81.3%)	45(82.6%)	*0.56 **0.000
SOFA	NA	NA	4.92 ± 3.1	8.13 ± 2.6	0,00
PCT (ng/mL)	NA	NA	8.6(1.8–24.0)	49.4(8.2–100.0)	<0.0001
WBC (x10^3^ cells/L)	NA	4.49 (3,07–5.43)	14.73 (8.1)	15.74 (10.9)	*0.56 **0.000
Total Bilirubin (umol/L)	NA		20.9 (12.9–58.1)	24.7(11.4–56.5)	0.98
Creatinine (mmol/L)	NA	76 (65.5–92.5)	94.0(72.0–144.5)	148.0(99.0–268.0)	*0.001
Lactate (mmol/L)	NA	NA	2.4(1.5–3.3)	4.5(2.6–7.0)	0.007
AST (IU/L)	NA	54.0 (21.0–100.0)	52.3 (27.0–102.5)	124.0 (42–216)	*<0.0001
ALT (IU/L)	NA	12.6 (9.6–30.5)	46.9 (27.2–82.0)	176.0 (34.0–190.0)	*<0.0001
Mortality rate	NA	NA	20 (40.5%)	32 (60.9%)	0.000
Blood culture positive	NA	NA	61 (78%)	32 (61.5%)	0.84
Gram negative	NA	NA	36 (46.2%)	22 (42.3%)	0.27
Gram positive	NA	NA	25 (32%)	10 (19.2%)	0.27

**Abbreviation**: DHF: Dengue Hemorrhagic Fever; HC: healthy control; PLT: platelets. AST and ALT: aspartate and alanine amino transferase; IU: international unit; NA: not applicable; NS: not significant. Values given are medians and range or mean ± SD. P values were calculated by Chi-squared tests, Mann-Whitney- Wilcoxon or Krusskal-Wallis tests where appropriate.

*: p Sepsis patients with Shock septic patient

**: p DHF-HC-Sepsis patients.

### High miRNA expression in sepsis patients

**Fig 1:**
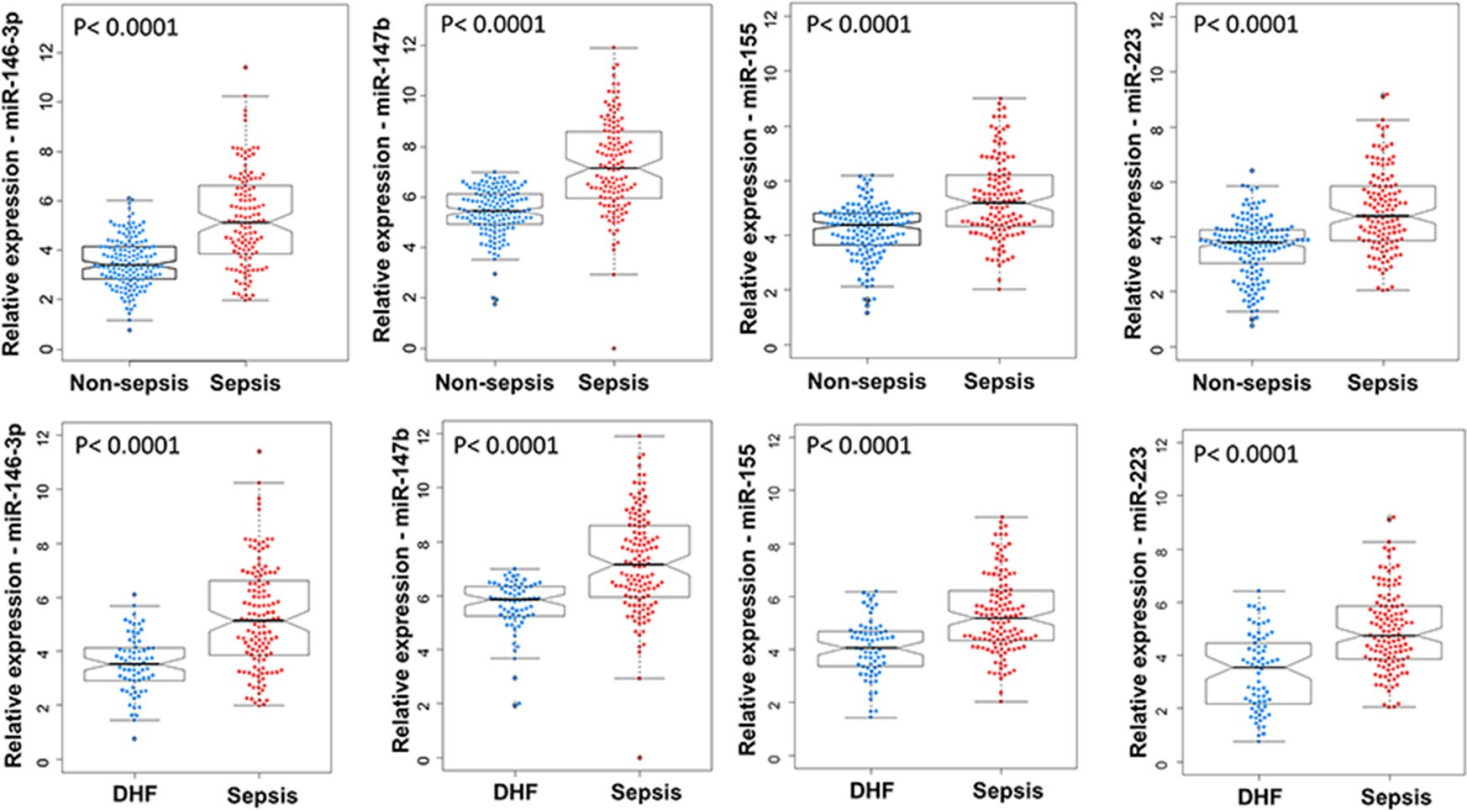
Expression of selected microRNAs is significantly elevated in sepsis patients compared to controls. Relative expression of individual miRNA (miR-146-3p, miR-147b, miR-155 and miR-223) in patients with and without sepsis. Non-sepsis group includes healthy controls (HC) and dengue hemorrhagic fever (DHF). P values were calculated by Wilcoxon tests.

**Fig 2:**
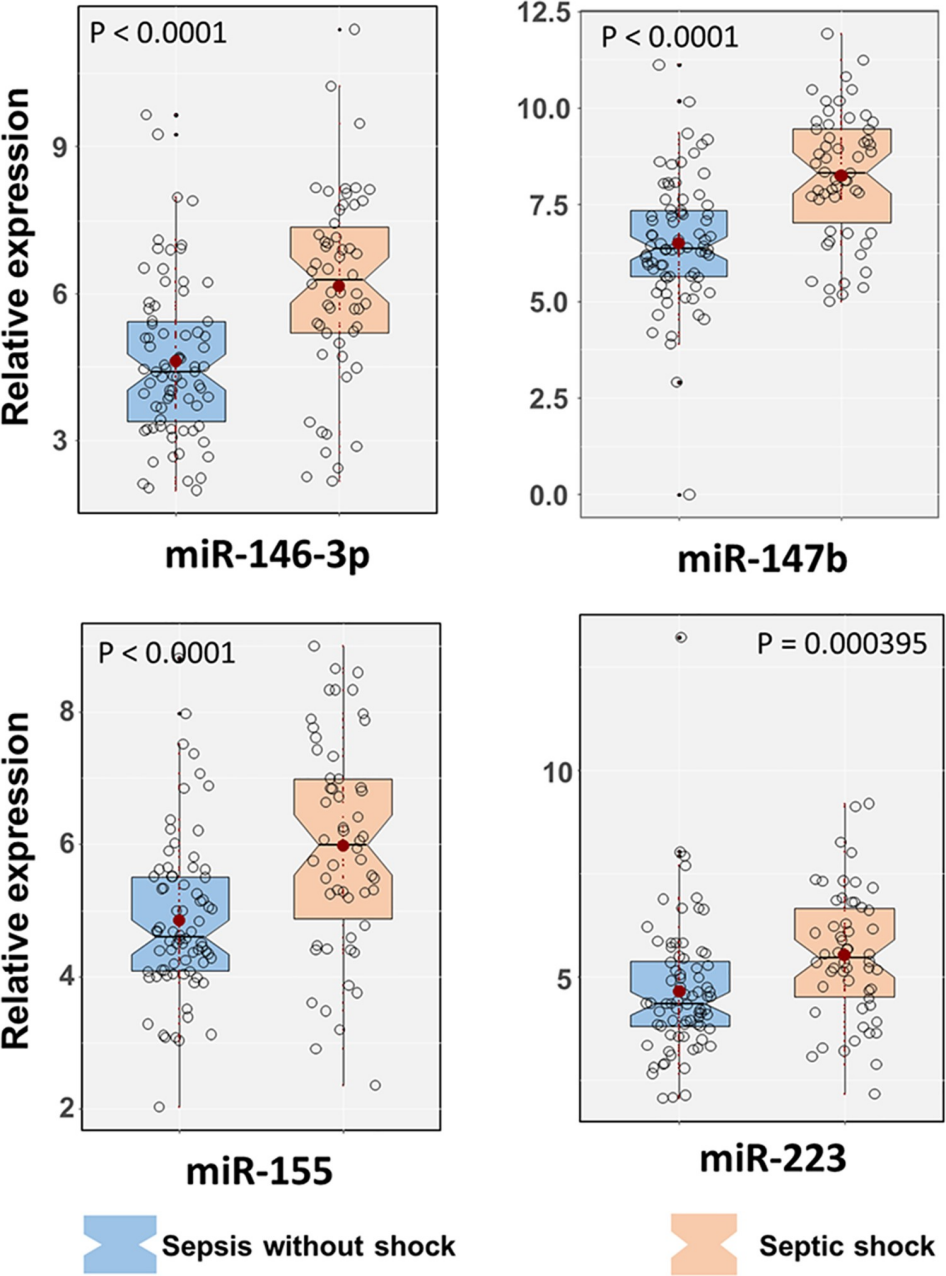
High expression of selected microRNAs is associated with septic shock. Relative expression of individual miRNA (miR-146-3p, miR-147b, miR-155 and miR-223) in sepsis patients with and without shock. P values were calculated by Wilcoxon tests.

The expression level of four inflammation-related miRNAs (miR-146-3p, miR-147b, miR-155 and miR-223) [11, 12, 17] was quantitively monitored for all study groups and normalized to internal control expression as previously described [20]. All four miRNAs (miR-146-3p, miR-147b, miR-155 and miR-223) had high expression in patient with sepsis compared to controls, DHF patients (Fig 1). The highest expression of four miRNAs studied was observed in patients with septic shock than other groups (Fig 2).

### Multiple miRNA expression and differential diagnosis in patients with sepsis

**Fig 3:**
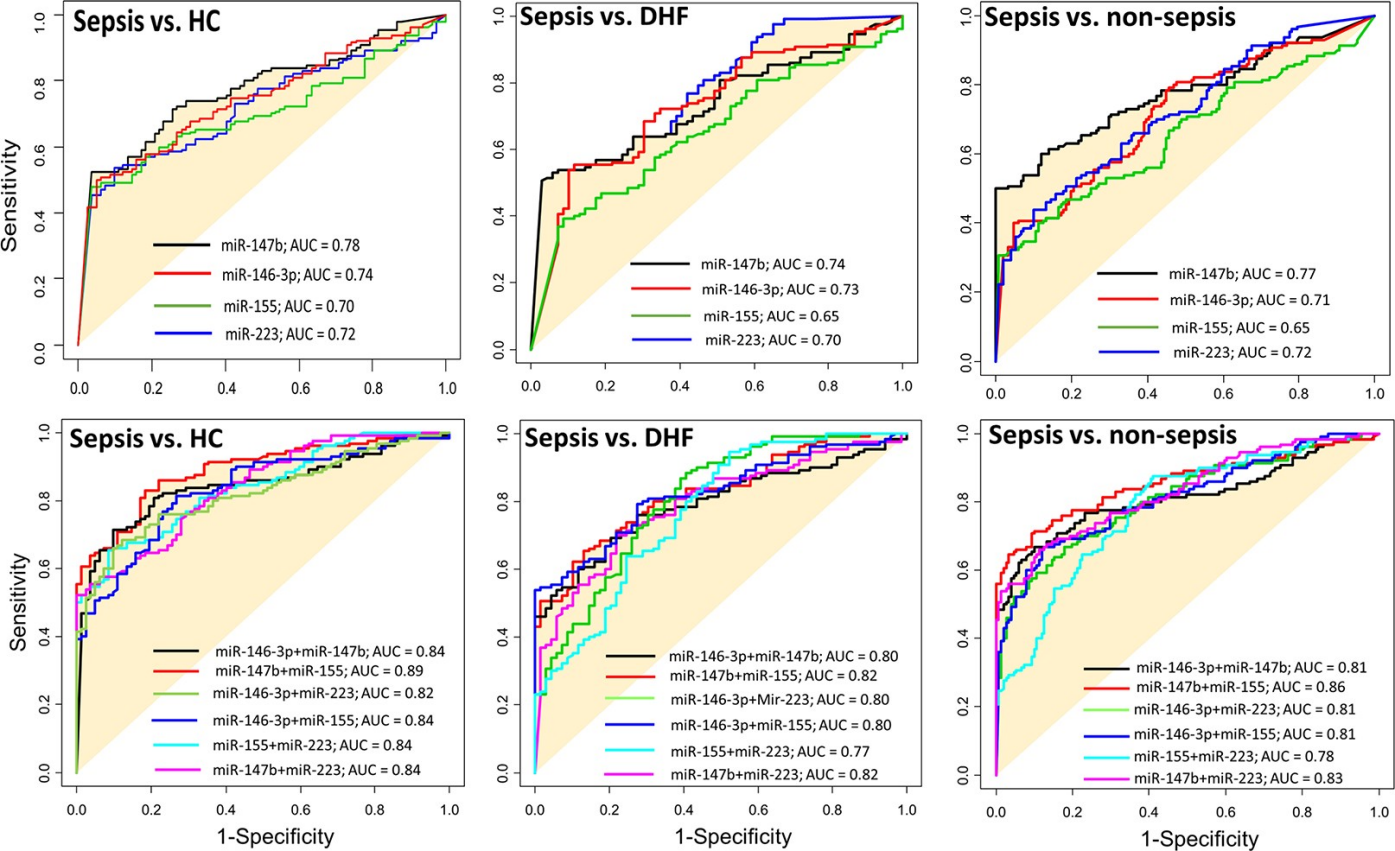
The value of miRNAs’ in differentiating diagnosis bacterial sepsis and non-sepsis groups. Diagnostic performance of the models built based on random forest models with single or double combination of miRNAs in differentiating sepsis patients from other groups HC, DHF and non-sepsis.

Similar to elevated PCT levels, individual miRNA expression (miR-146-3p, miR-147b, miR-155 and miR-223) showed a poor diagnostic performance and thus could not differentiate patients with sepsis and non-sepsis (AUC<0.8; Table 3 and Fig 3;). However, combined microRNA expression (miR-146-3p with miR-147b), (miR-147b with miR-155) and (miR-223 with miR-146-3p) showed a good diagnostic performance and could distinguish patients with sepsis from non-sepsis (AUC ≥ 0.8; Table 3 and Fig 3).

**Table 3:** The value of miRNA and PCT in differentiating diagnostics sepsis and non-sepsis and/or sepsis shock.

Variable(s)	Area Under the Curve (AUC)			
	Sepsis vs. HC	Sepsis vs. DHF	Sepsis vs. non-sepsis	Sepsis vs. shock sepsis
miR-146-3p	0.74 (*P*<0.05)	0.73 (*P*<0.05)	0.71 (*P*<0.05)	0.62 (*P*<0.05)
miR-147b	0.78 (*P*<0.05)	0.74 (*P*<0.05)	0.77 (*P*<0.05)	0.80 (*P*<0.05)
miR-155	0.70 (*P*<0.05)	0.65 (*P*<0.05)	0.65 (*P*<0.05)	0.66 (*P*<0.05)
miR-223	0.72 (*P*<0.05)	0.70 (*P*<0.05)	0.72 (*P*<0.05)	0.54 (*P*<0.05)
miR-146-3p & miR-147b combination	0.84 (*P*<0.05)	0.84 (*P*<0.05)	0.80 (*P*<0.05)	0.73 (*P*<0.05)
miR-147b & miR-155 combination	0.89 (*P*<0.05)	0.89 (*P*<0.05)	0.82 (*P*<0.05)	0.76 (*P*<0.05)
miR-155 & miR-223 combination	0.84 (*P*<0.05)	0.84 (*P*<0.05)	0.77 (*P*<0.05)	0.63 (*P*<0.05)
miR-223 & miR-146-3p combination	0.82 (*P*<0.05)	0.82 (*P*<0.05)	0.82 (*P*<0.05)	0.66 (*P*<0.05)
miR-146-3p & miR-155 combination	0.84 (*P*<0.05)	0.84 (*P*<0.05)	0.80 (*P*<0.05)	0.63 (*P*<0.05)
miR-147b & miR-223 combination	0.84 (*P*<0.05)	0.84 (*P*<0.05)	0.82 (*P*<0.05)	0.70 (*P*<0.05)
PCT	NA	NA	NA	0.69 (*P*<0.05)
miR-146-3p-3p +PCT	NA	NA	NA	0.77 (*P*<0.05)
miR-147b + PCT	NA	NA	NA	0.86 (*P*<0.05)
miR-155 + PCT	NA	NA	NA	0.77 (*P*<0.05)
miR-223+ PCT	NA	NA	NA	0.72 (*P*<0.05)

### miR147b and procalcitonin combination as potential predictor of sepsis shock

**Fig 4:**
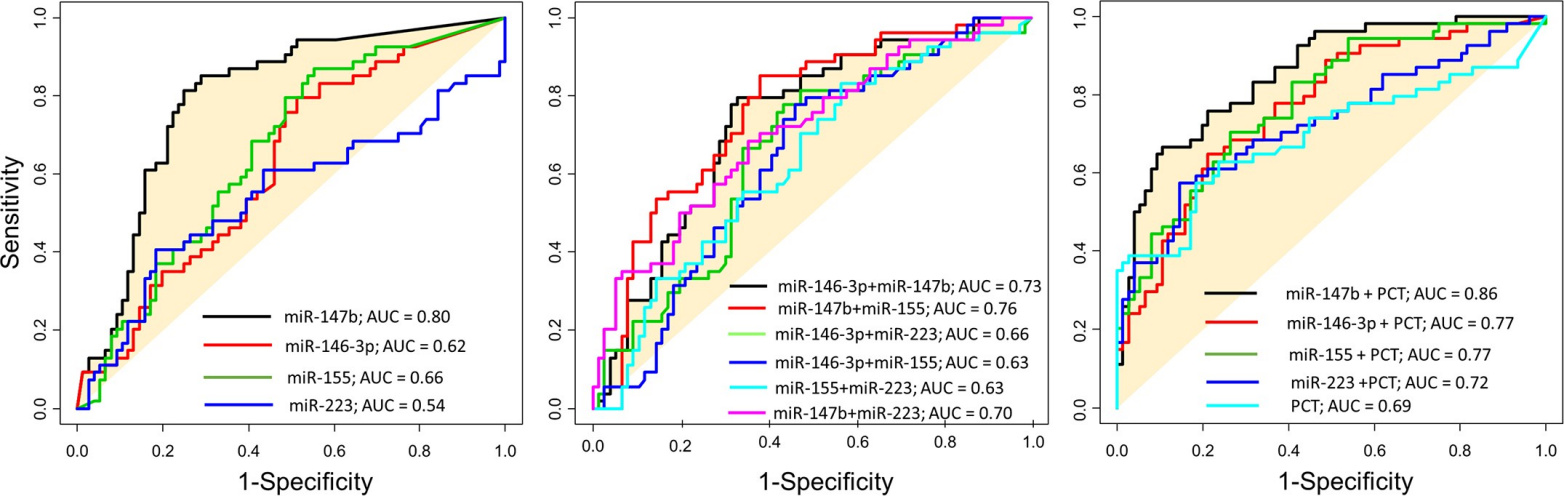
Combination of miR-147b and serological PCT can predict septic shock. Diagnostic performance of the models built based on random forest models with single or double combination of miRNAs or double combination of individual miRNA and PCT in differentiating septic shock patients from non-septic shock patients.

The use of miR-147b alone or the combination of miR-147b and PCT can significantly predict sepsis shock with a strong area under the curves (AUC) = 0.8 and 0.86, respectively (*P*<0.05) (Table 3 and Fig 4).

## Discussion

Bacterial sepsis requires rapid and prompt diagnosis, hence timely and appropriate antibacterial therapies can be initiated [21]. In clinical practice, bacterial sepsis overlaps with SIRS, which presents a diagnostics challenge [22–24]. In this study, we validated the expression of three selected miRNAs (miR-146-3p, miR-147b and miR-155) in a clinically defined cohort of patients with sepsis and compared the respective miRNA expression with healthy controls. Furthermore, the prognostic and diagnostic potential of these selected miRNAs are validated in a subset of patients with sepsis and septic shock using the respective miRNA expression levels and with PCT values.

Previous studies have already reported the role of miRNAs and the activation of NF-kappa B pathway, a pathological mechanism triggered by bacterial infections [10, 25, 26]. The inflammation-related microRNAs, such as miR-146-3p, miR-147b, miR-155 and miR-223 can be used as biomarkers to support the diagnostic establishment of bacterial sepsis. [10–16] and is evident that miR-223 is associated with platelet derived microparticles that modulate ICAM-1 expression on endothelial cells under septic environment [27]. Our current data confirm previous findings that the expression of the three microRNAs miR-146-3p, miR-155 and miR-223 is indeed increased in patients with bacterial sepsis [14, 16, 28, 29]. In particular, the expression of the inverstigated microRNAs are expresed higher in patients with sepsis than in DHF patients and/or healthy controls. Interestingly, miR-147b, which is induced by Toll-like receptors to regulate inflammatory responses, was significantly higher expressed in the peripheral blood of patients with bacterial sepsis [11]. The miR-147b was also shown to regulate vascular endothelial barrier function through ADAM15 expression during septic challenge [30]. Also, these results corroborate with serological PCT level in patients with sepsis. Aware of the fact that, early prediction of septic patients with shock helps clinicians to make the right indication for extraordinary intensive care, e.g. immediate antimicrobial and fluid resuscitation, which can somehow improve the survival rate [21, 31]. Our results provide evaluable evidence that miR-147b can be used independently or together with serological PCT to predict patients with septic shock. These findings are important because septic shock is the most severe subset of sepsis with a mortality rate of about 40% [24].

To date, several studies have proposed different microRNA panels to aid in the diagnosis of bacterial sepsis. However, no consensus has been reached on individual microRNA molecules and the data have not been recapitulated in different populations, and has not translated to clinical practice. In addition, the diagnostic panels described in previous studies require synthetic miRNAs such as mmu-miR-295 or cel-miR-39 as a reference for normalizing miRNA levels [14, 32]. This not only complicates laboratory work but is also difficult to standardize. While the use of synthetic miRNAs can provide better control accuracy in sample handling, it can ignore the natural degradation of endogenous molecules, as the synthetic miRNAs can acquire better stability than their internal counterparts such as miR-16 or U6 [20].

A limitation of this study is the validation of miRNAs in patients experiencing non-bacterial SIRS such as burns or acute pancreatitis, that may additionally signify the diagnostic value in distinguishing bacterial sepsis from SIRS patients.

In conclusion, the miR-147b may be used as a biomarker independently or in combination with PCT to support clinical diagnosis of sepsis and equally prognosis of patients with septic shock.
